# An End-to-End Deep Learning System for Gastrointestinal Bleeding Detection and Quantification in Wireless Capsule Endoscopy

**DOI:** 10.3390/diagnostics16132121

**Published:** 2026-07-07

**Authors:** Mujeeb Rahman Kanhira Kadavath, Aman Kitaz, Nour El Houda Benyahia, Shatha Hussein

**Affiliations:** Department of Biomedical Engineering, College of Engineering & Information Technology (CEIT), Ajman University, Ajman P.O. Box 346, United Arab Emirates; 202010457@ajmanuni.ac.ae (A.K.); 202010807@ajmanuni.ac.ae (N.E.H.B.); 202010809@ajmanuni.ac.ae (S.H.)

**Keywords:** gastro intestinal bleeding, wireless capsule endoscopy, machine learning, convolutional neural network, 2D-CNN, 3D-CNN, image segmentation, U-Net model, quantification of intestinal bleeding

## Abstract

**Background/Objectives**: Gastrointestinal bleeding is a critical finding in wireless capsule endoscopy (WCE), but manual examination of thousands of image frames is labor-intensive, time-consuming, and susceptible to missed lesions. This study aimed to develop and evaluate a comprehensive deep-learning framework for automated bleeding detection, localization, and quantitative assessment in WCE images. **Methods**: The proposed framework integrates three complementary deep-learning models: (i) a custom two-dimensional convolutional neural network (2D-CNN) for frame-level bleeding classification, (ii) a three-dimensional convolutional neural network (3D-CNN) for sequence-level analysis by exploiting temporal information from consecutive frames, and (iii) a U-Net architecture for pixel-level segmentation and bleeding-area quantification. The models were trained and evaluated using expert-annotated WCE datasets with pixel-level ground-truth masks. **Results**: The proposed 2D-CNN and 3D-CNN achieved excellent classification performance, with areas under the receiver operating characteristic curve (AUCs) of 0.9986 and 0.9971, respectively. The U-Net model achieved a Dice similarity coefficient of 0.93, an intersection-over-union (IoU) of 0.8677, and an overall segmentation accuracy of 97.25%. The integrated framework outperformed previously reported methods, demonstrating robust performance for bleeding detection, localization, and quantitative assessment. **Conclusions**: The proposed end-to-end deep-learning framework enables accurate automated bleeding detection, localization, and severity quantification in WCE images. By reducing the burden of manual image review, improving diagnostic consistency, and providing objective bleeding assessment, the framework has strong potential to support clinical decision-making and enhance gastrointestinal diagnostic workflows.

## 1. Introduction

The gastrointestinal (GI) tract is a continuous muscular organ responsible for digestion, nutrient absorption, and waste elimination [1,2,3]. Among GI disorders, gastrointestinal bleeding is one of the most clinically significant conditions, accounting for approximately 150 hospital admissions per 100,000 individuals annually and mortality rates ranging from 2% to 10% [4,5]. Early detection and localization of bleeding are therefore essential for timely clinical intervention and improved patient outcomes.

Conventional diagnostic techniques, including endoscopy and angiography, remain the clinical standard for evaluating GI bleeding. However, these procedures are invasive, often require sedation, and may not adequately visualize the entire small intestine [6,7]. Wireless Capsule Endoscopy (WCE) was developed to overcome these limitations. The swallowable capsule, approximately 26 × 11 mm in size, contains a miniature camera, light source, battery, and wireless transmitter, enabling non-invasive visualization of the entire GI tract, including regions inaccessible to conventional endoscopy [8,9,10]. During a typical examination, the capsule captures 2–6 images per second and may generate more than 50,000 images, making manual review labor-intensive, time-consuming, and susceptible to observer variability [10].

To alleviate this burden, numerous machine-learning (ML) and deep-learning approaches have been proposed for automated bleeding detection in WCE images. Early methods relied primarily on handcrafted color and texture features combined with conventional classifiers such as Support Vector Machines (SVMs) and clustering algorithms. For example, Deeba et al. achieved a Dice Similarity Coefficient (DSC) of 0.81 using an Automated GrowCut algorithm combined with SVM classification [11], while Yuan et al. reported 95.75% classification accuracy using color histograms and K-means clustering [12]. Although these approaches demonstrated the feasibility of automated bleeding detection, their performance was often sensitive to image variability and showed limited generalization across datasets [11,12,13,14,15].

Recent advances in deep learning, particularly Convolutional Neural Networks (CNNs), have significantly improved bleeding detection performance by automatically learning discriminative image features. CNN-based and U-Net-based models have achieved classification accuracies exceeding 95% and demonstrated promising segmentation performance for bleeding localization [16,17,18,19,20]. Rustam et al. introduced the Bleedy Image Recognizer (BIR), achieving a classification accuracy of 97.8% [21], while Aarushi et al. reported a precision of 98.11% and specificity of 98.55% using hybrid machine-learning techniques [22]. More recently, Bordbar et al. proposed a three-dimensional CNN (3D-CNN) capable of exploiting temporal information from consecutive WCE frames, highlighting the potential benefits of sequence-level analysis for bleeding detection [23].

Despite these advances, several important challenges remain. Most existing studies focus primarily on binary bleeding classification and provide limited information regarding the extent of visible bleeding. Consequently, the clinical interpretability of model outputs remains restricted. Furthermore, temporal information contained in consecutive WCE frames remains underutilized in many frameworks. The lack of patient-level metadata in publicly available datasets also complicates rigorous assessment of model generalizability and introduces the potential risk of data leakage. In addition, extensive reliance on synthetic augmentation to address class imbalance may not fully reflect real-world clinical variability [24,25,26].

Motivated by these limitations, this study proposes a comprehensive deep-learning framework for automated gastrointestinal bleeding analysis in WCE images. The framework integrates a custom 2D-CNN for frame-level classification, a 3D-CNN for sequence-level classification using temporal information, and a U-Net architecture for pixel-level segmentation of bleeding regions. The resulting segmentation masks are further utilized to estimate the visible bleeding area, providing an objective image-based measure of bleeding burden. The proposed framework was evaluated using three publicly available WCE datasets to assess its robustness and generalizability.

The main contributions of this study are summarized as follows:

- Development of lightweight custom 2D-CNN, 3D-CNN, and U-Net architectures for bleeding analysis at frame, sequence, and pixel levels.
- Organization and preparation of WCE datasets suitable for both classification and segmentation tasks.
- Introduction of quantitative bleeding-area estimation as an image-based measure of visible bleeding burden.
- Evaluation using three publicly available WCE datasets to enhance robustness, diversity, and generalizability.
- Generation of clinically interpretable outputs that extend beyond binary detection and support diagnostic decision-making.

## 2. Materials and Methods

Four publicly available, de-identified Wireless Capsule Endoscopy (WCE) datasets were used to develop and validate the proposed framework.

- Dataset 1 (Kvasir-Capsule): A large-scale WCE repository containing approximately 4.7 million frames. For this study, 446 bleeding and 421 normal temporally continuous frames were selected (867 frames total) to support sequence-based analysis using the 3D-CNN model [27].
- Dataset 2 (Red Lesion Endoscopy—Set 1): A dataset comprising 3295 frames (320 × 320 pixels) with expert-annotated bleeding masks. These masks were used to train and evaluate the U-Net model for pixel-level bleeding localization and quantification [28].
- Dataset 3 (Turkey Hospital Muzaffargarh): A collection of 226 WCE images (113 bleeding and 113 non-bleeding) with a resolution of 1288 × 964 pixels, used for frame-level bleeding detection [29].
- Dataset 4 (Combined Dataset): A merged dataset containing 4388 frames (1690 bleeding and 2698 non-bleeding), created by combining Datasets 1–3. This dataset was used for developing the 2D-CNN classification model and provided increased diversity and variability for training.

**Figure 1:**
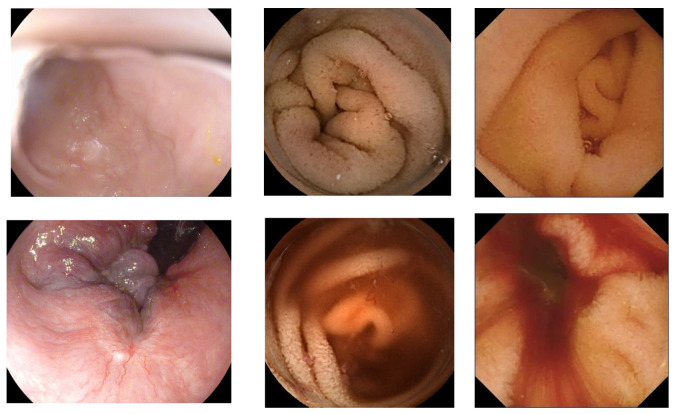
Representative WCE frames from Dataset 4, illustrating variations in image resolution, illumination, and mucosal appearance: The top row shows normal frames, while the bottom row shows bleeding frames.

Table 1 provides an overview of the datasets used in this study, while Figure 1 presents representative sample images from Dataset 4, the combined dataset.

### 2.1. Overview of the Proposed Bleeding Detection and Quantification Framework

This section presents the proposed gastrointestinal bleeding analysis framework, which integrates custom 2D-CNN and 3D-CNN models for automated bleeding detection and a U-Net model for bleeding localization and quantification. Dataset 1 was used to develop the 3D-CNN model, Dataset 2 was used for U-Net-based bleeding segmentation, and the composite Dataset 4 was used to train and evaluate the 2D-CNN model.

**Figure 2:**
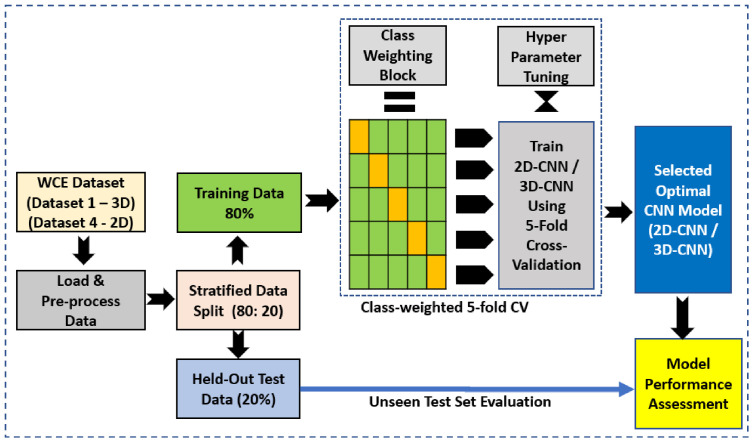
Overview of the proposed 2D-CNN and 3D-CNN bleeding detection framework.

As shown in Figure 2, the 2D-CNN and 3D-CNN models share a common preprocessing pipeline. Images are loaded, unreadable frames are removed, and the remaining images are converted to RGB format, resized, normalized, and assigned their corresponding class labels. The 2D-CNN processes individual frames independently, whereas the 3D-CNN operates on previously constructed 3D image sequences formed from consecutive frames, enabling the model to capture temporal and contextual information across adjacent images.

Following preprocessing, the datasets are partitioned into training and testing sets using a stratified 80:20 split. The training set (80%) is used for 5-fold stratified cross-validation, while the remaining 20% is reserved as an independent test set. To address class imbalance and improve model generalization, class-weighted loss functions are employed during training. In each cross-validation fold, one subset is used for validation and the remaining subsets are used for training. Model weights are reinitialized at the start of every fold to ensure independent evaluation. The average performance across all folds is used to assess model robustness [30]. Finally, the best-performing model is retrained and evaluated on the unseen held-out test set.

**Figure 3:**
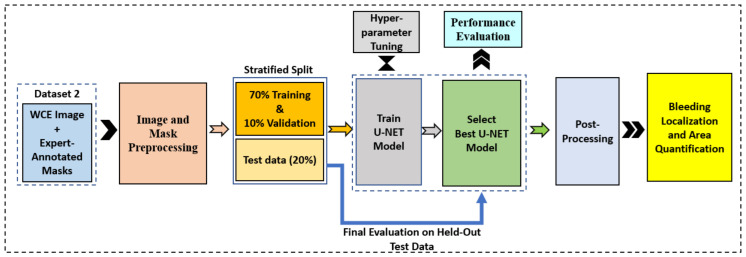
Overview of the proposed U-Net-based bleeding localization and quantification framework.

The U-Net segmentation framework, given in Figure 3, utilizes WCE images paired with expert-annotated masks. Images and masks undergo preprocessing, including loading, resizing, normalization, and mask alignment. The dataset is then stratified into training (70%), validation (10%), and testing (20%) subsets to ensure balanced representation of bleeding and non-bleeding samples. The U-Net model is trained with hyperparameter tuning and validated using the dedicated validation set. The optimal model is subsequently evaluated on the unseen test set. Finally, post-processing is applied to refine segmentation masks, extract lesion contours, and compute bleeding-area percentages for quantitative assessment. Detailed model architectures and design considerations are presented in the following sections.

#### 2.1.1. Architecture of the 2D-CNN

**Figure 4:**
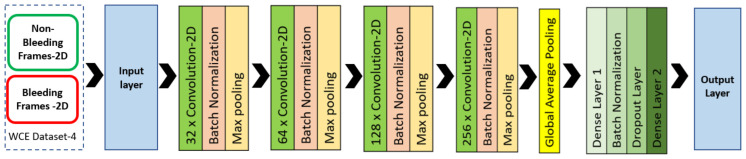
Architecture of the proposed 2D-CNN model for bleeding frame classification.

The architecture of the 2D-CNN detection model is presented in Figure 4. The 2D-CNN model takes individual bleeding and non-bleeding WCE frames from Dataset-4 as input. It consists of four convolutional blocks with 32, 64, 128, and 256 filters, each followed by Batch Normalization and Max-Pooling to extract spatial features. A Global Average Pooling layer condenses these features before two dense layers with Batch Normalization and Dropout for regularization. The final output layer performs binary classification of bleeding versus non-bleeding frames.

We initialized the model using preset hyperparameters—such as learning rate, batch size, dropout rate, epochs, and kernel dimensions—derived from previous research, and then refined them through iterative experimentation to achieve optimal validation performance. The final 2D-CNN model comprises 423,875 parameters (1.62 MB), including 422,657 trainable (1.61 MB) and 1216 non-trainable parameters (4.75 KB), with the optimizer adding 2 parameters (12 B).

#### 2.1.2. Architecture of the 3D-CNN

**Figure 5:**
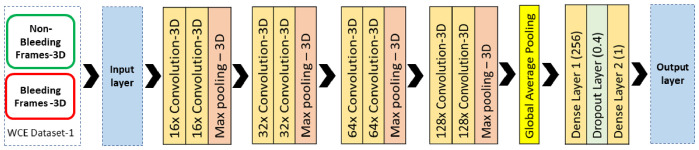
Architecture of the proposed 3D-CNN model for bleeding frame classification.

In Figure 5, the proposed 3D-CNN model processes short sequences of consecutive frames from Dataset 1, enabling the extraction of both spatial and temporal information relevant to gastrointestinal bleeding detection. The architecture consists of four sequential 3D convolutional blocks with 16, 32, 64, and 128 filters, respectively. Each convolutional block is followed by a 3D max-pooling layer, which progressively reduces the spatial–temporal dimensions while preserving salient features and reducing computational complexity. Following feature extraction, a Global Average Pooling layer is employed to aggregate the learned spatiotemporal features into a compact feature representation, significantly reducing the number of trainable parameters compared with conventional flattening operations. The resulting feature vector is passed through a fully connected layer containing 256 neurons, followed by a dropout layer with a rate of 0.4 to mitigate overfitting and improve generalization. Finally, a single sigmoid-activated output neuron performs binary classification to determine whether the input frame sequence corresponds to a bleeding or non-bleeding event.

The final 3D-CNN model comprises 300,257 parameters (1.15 MB), including 299,777 trainable (1.14 MB) and 480 non-trainable parameters (1.88 KB). This lightweight architecture reduces computational complexity and memory requirements while mitigating the risk of overfitting, thereby enabling efficient learning of spatial–temporal bleeding patterns from consecutive WCE frames.

#### 2.1.3. Architecture of the U-NET

**Figure 6:**
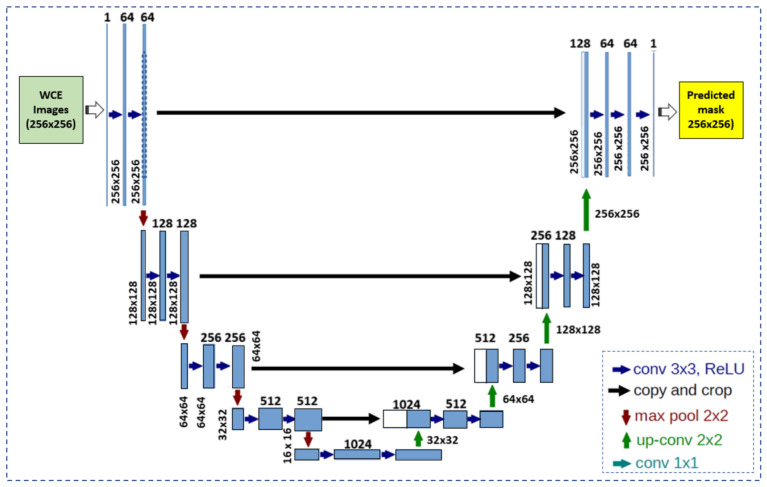
Architecture of the Proposed U-Net Model for Bleeding Segmentation.

Figure 6 illustrates the U-Net architecture employed for bleeding segmentation in WCE images. The model adopts a symmetric encoder–decoder structure designed to capture both contextual and spatial information. In the encoder path, the input image (256 × 256 × 1) is processed through successive 3 × 3 convolutional layers with ReLU activation, followed by 2 × 2 max-pooling operations. This progressively reduces the spatial resolution while increasing the feature depth from 64 to 1024 channels, enabling the extraction of increasingly abstract image representations.

The decoder path mirrors the encoder architecture. At each decoding stage, 2 × 2 up-convolution operations restore the spatial resolution, and the resulting feature maps are concatenated with the corresponding encoder features through skip connections. These skip connections facilitate multi-scale feature fusion by preserving fine-grained spatial details that may otherwise be lost during down-sampling, thereby improving localization accuracy and boundary delineation [31,32].

Each up-sampling stage is followed by additional 3 × 3 convolutional layers with ReLU activation to refine the reconstructed feature maps. A final 1 × 1 convolution layer generates a 256 × 256 segmentation mask representing the predicted bleeding region. By combining high-level contextual information with low-level spatial features, the U-Net architecture is well-suited for accurate medical image segmentation.

For model development, the training set was used for network optimization, while a validation set was employed for hyperparameter tuning and model selection. The best-performing U-Net model, determined using validation metrics, was subsequently evaluated on an independent held-out test set comprising 20% of the dataset. This test set remained completely unseen during training and validation, ensuring an unbiased assessment of segmentation performance.

### 2.2. Contouring and Bleeding Area Calculation

After selecting the best-performing U-Net model, segmentation masks were generated for all test images and binarized using a threshold of 0.5. This threshold was selected because it is the standard decision threshold for binary segmentation models employing a sigmoid output layer and is widely adopted in the U-Net literature. Furthermore, a threshold optimization study performed on the validation set showed that although a higher threshold (~0.9999) achieved a slightly higher Dice score (0.811 vs. 0.793), the improvement was modest (approximately 1.8 percentage points). Therefore, the conventional threshold of 0.5 was retained to maintain consistency, reproducibility, and a favorable precision–recall tradeoff.

For each test sample, the Intersection over Union (IoU) between the predicted and ground-truth masks was computed [33]. Representative segmentation results and summary statistics, including the minimum, maximum, and mean IoU values, were subsequently reported.

The binary prediction masks were then converted to 8-bit format, and contour extraction was performed using the ‘cv2.findContours‘ function to identify connected bleeding regions. The enclosed contour areas were calculated and expressed as a percentage of the total image area, providing an objective image-based measure of visible bleeding burden.

For qualitative evaluation, a random subset of test samples was visualized with ground-truth and predicted contours overlaid on the original WCE frames, together with their corresponding bleeding-area percentages.

### 2.3. Performance Evaluation Metrics

The following metrics were adopted to assess the performance of the classification and segmentation models:

(a) Accuracy (ACC): The portion of correctly identified cases to the total number of tested cases.
  $$ACC=\frac{TP+TN}{Total\;Cases\;Tested}$$
(b) Precision (P): Assesses the frequency with which a machine learning model accurately identifies the positive class.
  $$P=\frac{TP}{TP+FP}$$
(c) Recall (R): Also known as sensitivity or True Positive Rate (TPR), this metric evaluates how effectively the model detects actual positive cases.
  $$R=\frac{TP}{TP+FN}$$
(d) Specificity (S): Also known as the True Negative Rate (TNR), it evaluates how accurately the model detects actual negative cases.
  $$S=\frac{TN}{TN+FP}$$
(e) F1 score (F1): A metric that measures a test’s accuracy by computing the harmonic mean of precision and recall. The F1 score ranges from 0, indicating the lowest accuracy, to 1, representing the highest accuracy.
  $$F1=\frac{2*P*R}{P+R}$$

In this context, True Positives (TP) are situations where positive cases are correctly identified as positive. True Negatives (TN) are scenarios where negative cases are accurately recognized as negative. False Negatives (FN) occur when positive cases are incorrectly labeled as negative, while False Positives (FP) are cases where negative instances are wrongly classified as positive [30].

Intersection over Union (IoU) is a commonly used assessment metric for segmentation tasks; it is often referred to as the Jaccard index. By measuring the extent of overlap between the model’s predictions and the ground truth for the same object, this approach assesses the accuracy of segmentation [34].

$$IoU=\frac{\left|A\cap B\right|}{\;|A\cup B|\;}$$

The Receiver Operating Characteristic (ROC) curve illustrates the trade-off between the True Positive Rate (TPR) and False Positive Rate (FPR) across various thresholds, offering an overall view of model performance [35]. The Area Under the Curve (AUC) provides a single, threshold-independent measure of class discrimination, where higher values indicate better performance. Compared to accuracy, AUC is more robust for imbalanced datasets, while accuracy suits balanced data with fixed thresholds [36]. Hence, AUC was adopted as one of the key performance metrics in this study.

### 2.4. Model Development Environment

The proposed models were developed using Python 3.11 in a Jupyter Notebook environment. Experiments were conducted on a Dell XPS workstation equipped with an Intel Core Ultra 7-155H processor (Dell, 16 cores, up to 4.8 GHz), 64 GB RAM, 2 TB SSD storage, and an NVIDIA GeForce RTX 4060 GPU, providing sufficient computational resources for deep-learning model training and evaluation. Google Colab was additionally used for selected experiments and software environment management.

## 3. Results

This section reports the performance of the 2D-CNN, 3D-CNN, and U-Net models across the classification and segmentation tasks on WCE images. The evaluation focuses on the models’ ability to identify gastrointestinal bleeding frames and accurately segment bleeding regions.

### 3.1. Performance of 2D-CNN Model

For the 2D-CNN model, the dataset of 4388 WCE images was divided using a stratified 80:20 split, yielding 3509 images for training and validation and 879 images for held-out testing. The training subset was further evaluated using 5-fold cross-validation, with each fold comprising 2808 training samples and 702 validation samples, enabling a consistent and balanced assessment of the model’s classification performance.

**Figure 7:**
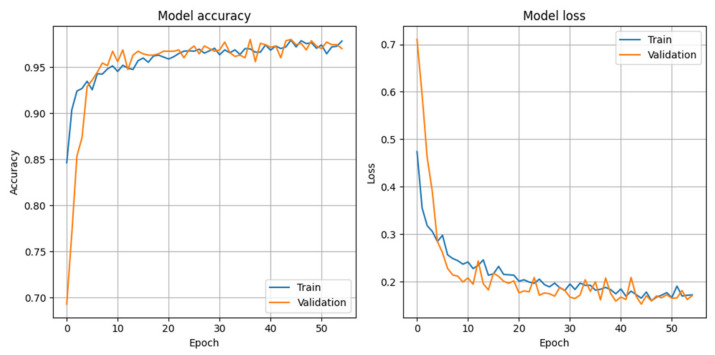
Learning Curves of the 2D-CNN Model: Training vs. Validation Accuracy (**left**) and Loss (**right**).

Figure 7 illustrates the model’s learning behavior during training by presenting the training and validation accuracy and loss curves, highlighting the best-performing epoch. Table 2 summarizes the fold-wise performance metrics of the 2D-CNN along with the mean and standard deviation.

**Figure 8:**
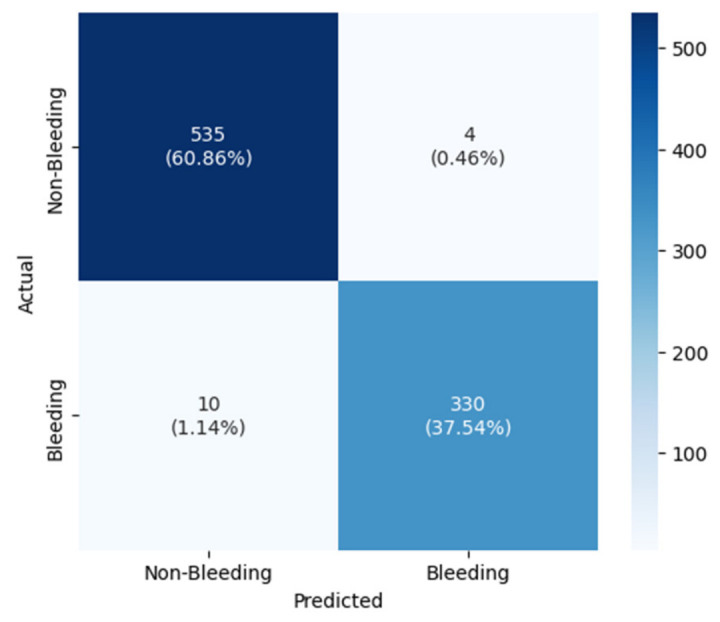
Confusion Matrix of the Proposed 2D-CNN Model Evaluated on the Held-Out Test Dataset.

**Figure 9:**
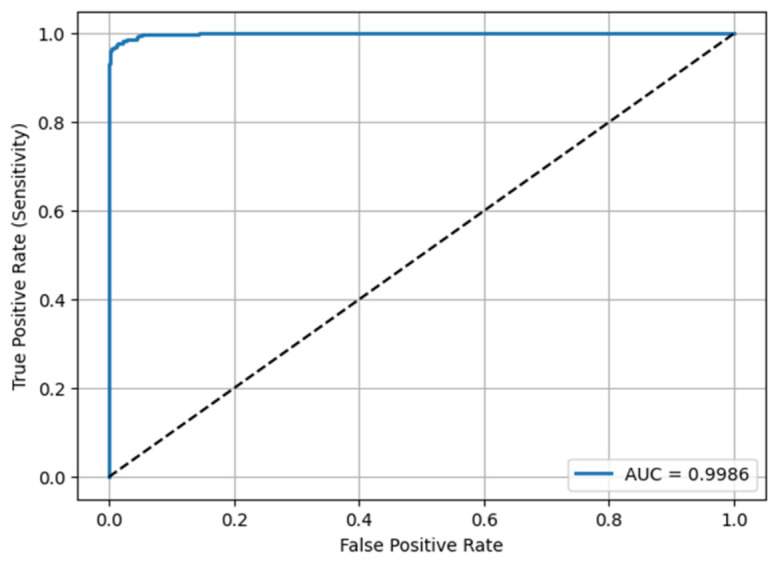
ROC Curve of the Proposed 2D-CNN Model Evaluated on the Held-Out Test Dataset.

Figure 8 presents the confusion matrix obtained on the held-out test dataset, while Figure 9 shows the corresponding ROC curve and AUC. The proposed 2D-CNN model achieved an accuracy of 98.41%, with 330 true positives, 535 true negatives, 4 false positives, and 10 false negatives. The high precision (98.88%) and recall (97.06%) indicate reliable detection of bleeding frames with few false alarms. An AUC of 0.9986 further demonstrates excellent discrimination between bleeding and non-bleeding frames. The test-set performance was consistent with the cross-validation results, suggesting good generalization and limited overfitting.

**Figure 10:**
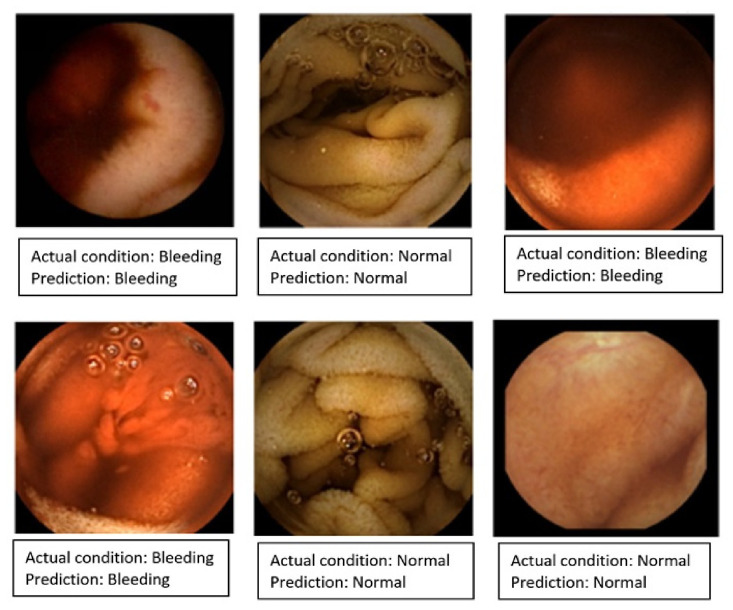
Representative 2D-CNN Classification Outputs Showing Correctly Predicted Bleeding and Normal Cases.

Figure 10 presents representative outputs of the 2D-CNN model on unseen bleeding and non-bleeding test images. For each sample, the figure displays the actual ground-truth condition (bleeding or normal) alongside the model’s predicted label, illustrating correct classifications across different visual appearances of WCE frames.

### 3.2. Performance of 3D-CNN Model

For the 3D-CNN model a total of 867 WCE frames were converted into 64 × 64 × 64 volumes. A stratified 80:20 split resulted in 693 volumes for training and validation and 174 volumes reserved as a held-out test set. The training portion was further assessed using 5-fold cross-validation, with each fold comprising approximately 554 training samples and 139 validation samples. The held-out test set (174 volumes) remained fixed for final performance evaluation.

**Figure 11:**
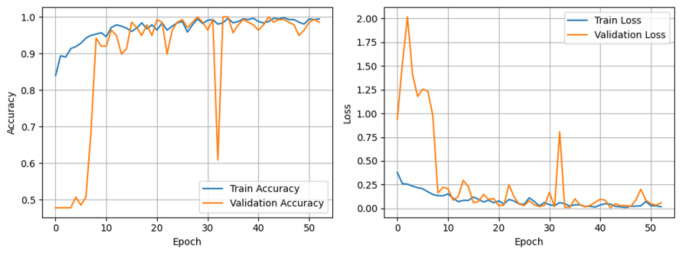
Learning Curves of the 3D-CNN Model: Training vs. Validation Accuracy (**left**) and Loss (**right**).

Figure 11 illustrates the model’s learning behavior during training by presenting the training and validation accuracy and loss curves, highlighting the best-performing epoch. Table 3 summarizes the fold-wise performance metrics of the 3D-CNN along with the mean and standard deviation.

**Figure 12:**
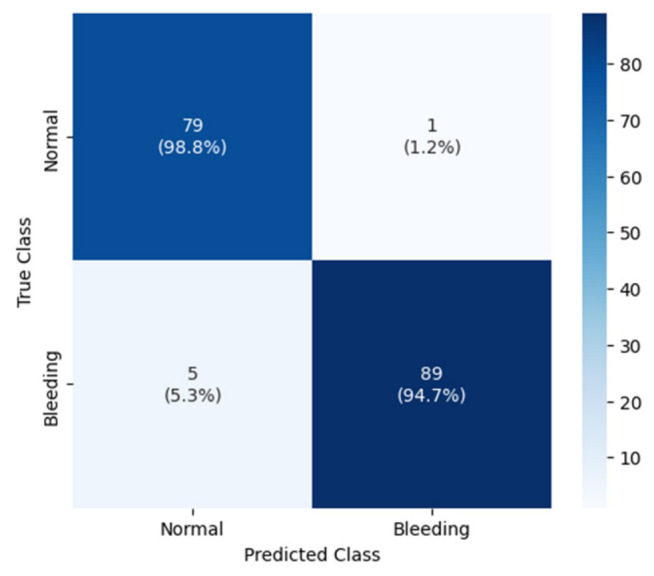
Confusion Matrix of the Proposed 3D-CNN Model Evaluated on the Held-Out Test Dataset.

**Figure 13:**
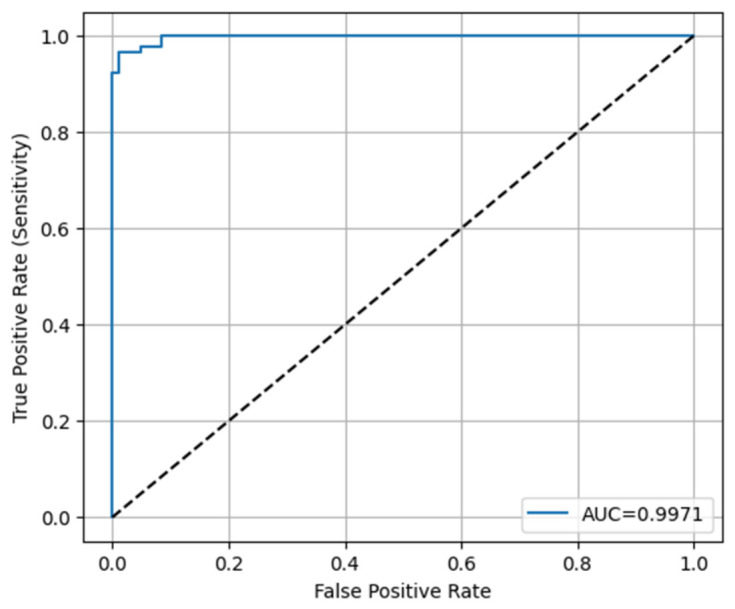
ROC Curve of the Proposed 3D-CNN Model Evaluated on the Held-Out Test Dataset. The blue curve represents the ROC performance of the proposed model, while the black dashed diagonal line represents the performance of a random classifier (AUC = 0.5).

Figure 12 presents the confusion matrix obtained on the held-out test dataset, summarizing the classification performance of the proposed 3D-CNN model. Figure 13 shows the corresponding ROC curve and AUC, illustrating the model’s discriminative capability across different classification thresholds. The model achieved an accuracy of 96.55%, precision of 98.89%, recall of 94.68%, and F1-score of 96.74%. The corresponding confusion matrix comprised 89 true positives, 79 true negatives, 1 false positive, and 5 false negatives. Furthermore, an AUC of 0.9971 was obtained, indicating excellent discrimination between bleeding and non-bleeding frames.

**Figure 14:**
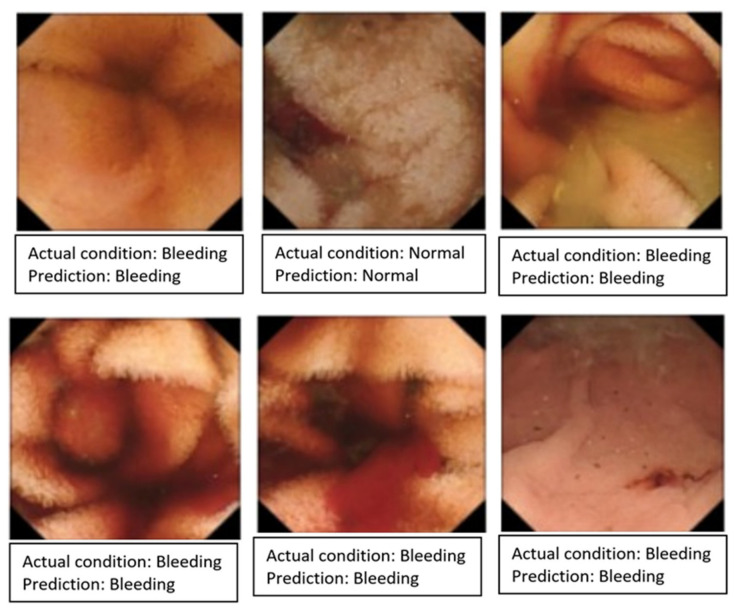
Representative 3D-CNN classification outputs showing correctly predicted bleeding and normal cases.

Figure 14 presents representative outputs of the 3D-CNN model on unseen bleeding and non-bleeding test images, showing each frame with its ground-truth label and the corresponding model prediction.

### 3.3. Performance of Segmentation and Bleeding Quantification Model

**Figure 15:**
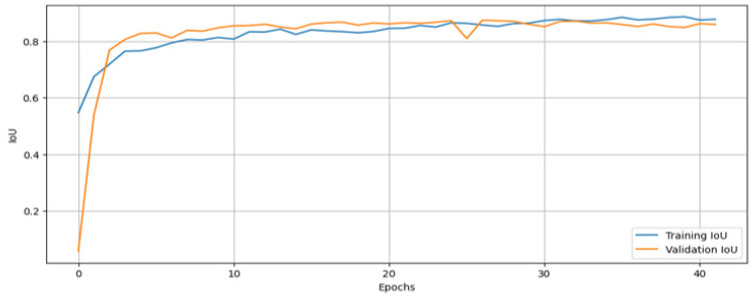
Learning curves of the proposed U-Net model showing training and validation IoU.

For the U-Net segmentation experiments, the dataset comprised 3295 WCE images and their corresponding ground-truth masks. A stratified 70:10:20 split was applied, resulting in 2306 training samples, 329 validation samples, and 659 test samples. The input image dimensions were (256 × 256 × 3), while the corresponding mask dimensions were (256 × 256 × 1). Figure 15 presents the training and validation IoU curves, illustrating the convergence behavior of the model during training. To determine the optimal segmentation threshold, multiple threshold values were evaluated on the validation set and their corresponding Dice coefficients were analyzed.

**Figure 16:**
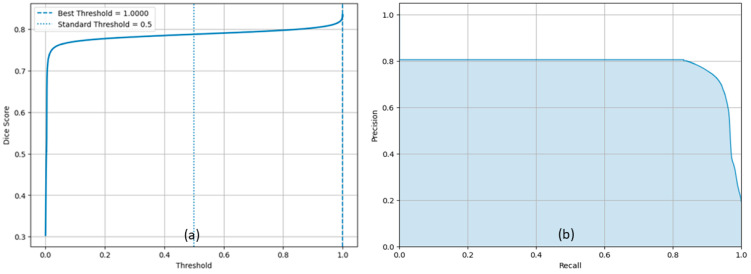
Threshold optimization analysis of the U-Net segmentation model. (**a**) Dice score as a function of the segmentation threshold. (**b**) Precision–Recall curve on the independent test set.

To determine the optimal segmentation threshold for the U-Net model, multiple threshold values were evaluated on the validation set. As shown in Figure 16a, the Dice score increased from 0.793 at the conventional threshold of 0.5 to 0.810 at the optimal threshold of 0.9999. Because the improvement was marginal, a threshold of 0.5 was retained for subsequent analyses. Figure 16b shows the Precision–Recall (PR) curve on the independent test set, achieving an Average Precision (AP) of 0.78. Using the standard threshold, the final test-set evaluation achieved a Dice coefficient of 0.9325.

The U-Net model demonstrated strong segmentation performance on the test dataset, achieving a Dice coefficient of 0.9325, precision of 0.9524, recall of 0.9164, and F1-score of 0.9292. The model also achieved an overall accuracy of 0.9750 with a test loss of 0.1289, indicating robust segmentation performance. At the pixel level, bleeding-pixel recall and background-pixel accuracy reached 92.93% and 91.49%, respectively, demonstrating accurate identification of bleeding regions with limited pixel-level misclassification.

**Figure 17:**
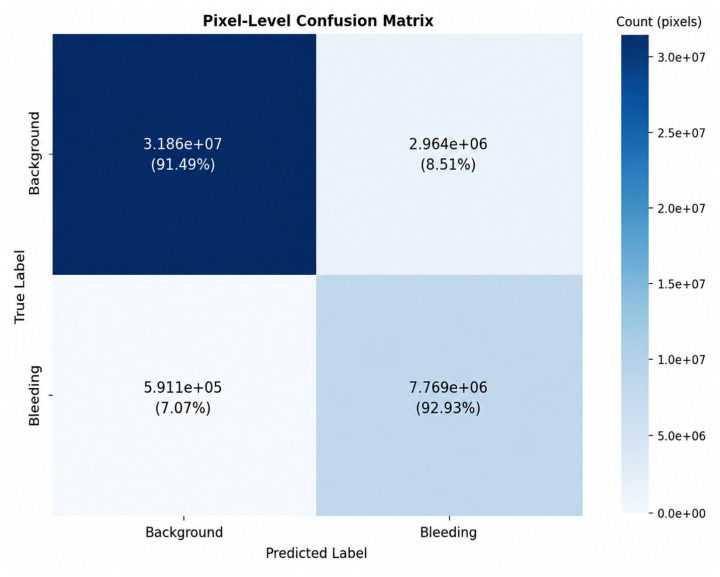
Pixel-Level Confusion Matrix of the U-Net Segmentation Model on the Test Dataset.

Figure 17 shows the pixel-level confusion matrix, confirming the U-Net model’s high segmentation accuracy, with 91.49% correct background classification and 92.93% correct bleeding pixel detection.

**Figure 18:**
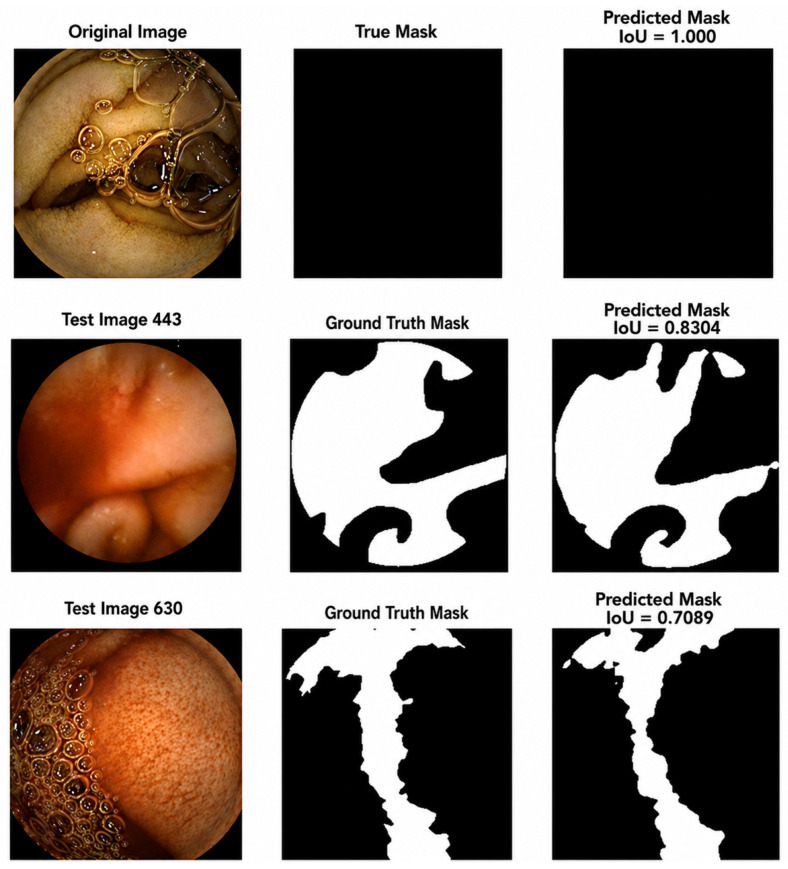
Representative U-Net segmentation results showing original WCE images, ground-truth masks, predicted masks, and IoU scores.

Figure 18 presents representative U-Net segmentation outputs, with each row displaying the original WCE image together with its ground-truth mask, the predicted mask, and the corresponding IoU score. The examples include two randomly selected bleeding cases and one non-bleeding case from the test set.

**Figure 19:**
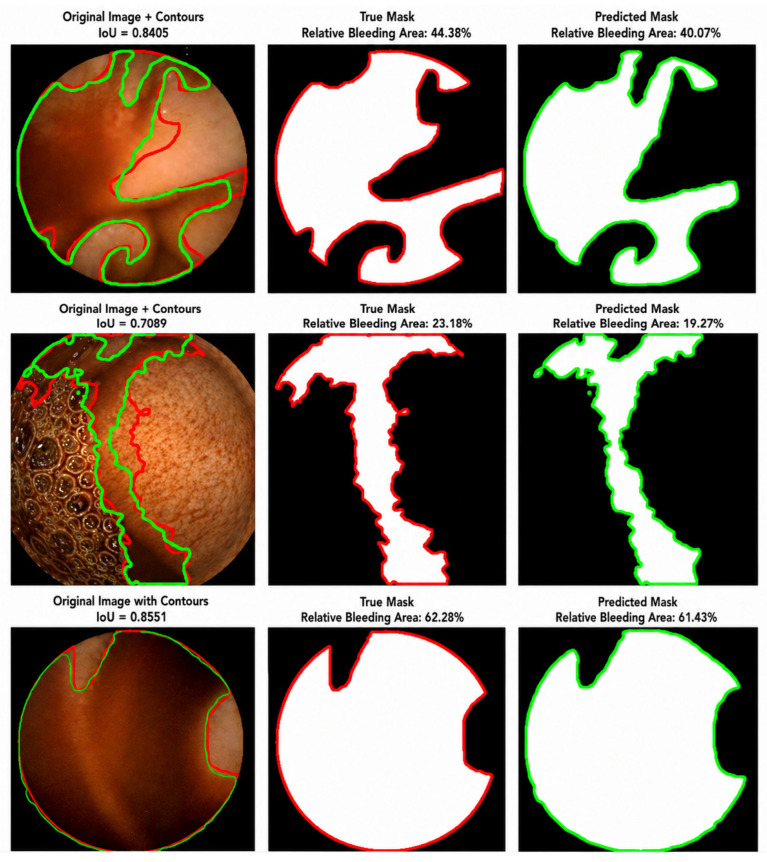
Contour-Based Bleeding Localization and Quantification Results Using Expert-Annotated (Red) and Predicted (Green) Boundaries.

Figure 19 presents illustrative examples of the U-Net segmentation results. Each row shows the original WCE image overlaid with expert-annotated contours (red) and predicted contours (green), followed by the corresponding ground-truth and predicted masks. The bleeding-area percentages derived from the expert-annotated and predicted masks are also reported for each sample. The estimated bleeding-area percentages derived from predicted masks closely matched those obtained from expert annotations.

## 4. Discussion

### 4.1. Interpretation of the 2D-CNN Model Performance

As shown in Table 2, the proposed 2D-CNN model demonstrated consistently strong performance across all cross-validation folds, achieving a mean AUC of 0.9978 ± 0.0010 and a mean accuracy of 97.47%. The low variability across folds and the close agreement between the training and validation curves (Figure 7) indicate stable learning and good generalization with minimal overfitting. The high precision, recall, and F1-score further demonstrate reliable detection of bleeding frames while maintaining a low false-alarm rate.

Evaluation on the held-out test set confirmed the robustness of the model. An AUC of 0.9986 and only 14 misclassifications (4 false positives and 10 false negatives) indicate excellent discrimination between bleeding and non-bleeding frames. The high precision (98.88%) and recall (97.06%) demonstrate reliable identification of bleeding events with few false detections. Representative examples in Figure 10 further illustrate the model’s ability to accurately classify WCE frames with diverse visual appearances.

### 4.2. Interpretation of the 3D-CNN Model Performance

The proposed 3D-CNN model, designed to exploit spatiotemporal information across short WCE sequences, demonstrated excellent classification performance. As shown in Figure 11, the training and validation curves exhibited stable convergence with minimal divergence, indicating good generalization despite the increased complexity of volumetric data.

Across the five cross-validation folds (Table 3), the model achieved a mean AUC of 0.9971 and a mean accuracy of 97.98%, reflecting strong discriminative capability and consistent learning. The close agreement between precision and recall resulted in a balanced F1-score of 0.9803, indicating reliable detection performance. Notably, these results were achieved using a substantially smaller dataset than that used for the 2D-CNN, suggesting that temporal information can provide valuable discriminative cues for bleeding detection.

Evaluation on the held-out test set further confirmed the robustness of the model. An AUC of 0.9971 and only five misclassifications (1 false positive and 5 false negatives) demonstrate excellent discrimination between bleeding and non-bleeding sequences. Representative examples in Figure 14 further illustrate the model’s ability to accurately classify previously unseen WCE sequences.

### 4.3. Interpretation of the UNET Segmentation Model Performance

As shown in Figure 15, the U-Net model exhibited stable learning behavior, with smooth convergence of the training and validation curves and no evident signs of overfitting. The model achieved strong segmentation performance, attaining a Dice coefficient of 0.9325 and an IoU of 0.8677, indicating excellent agreement between the predicted and ground-truth masks. The high precision and recall further demonstrate reliable identification of bleeding regions while maintaining a low rate of false detections.

Qualitative results shown in Figure 18 and Figure 19 further confirm the robustness of the segmentation model. The U-Net successfully localized bleeding regions across diverse lesion shapes and sizes, including irregular, elongated, and well-defined bleeding patterns. The close agreement between expert-annotated and predicted contours, as well as the corresponding bleeding-area estimates, demonstrates the model’s ability to accurately delineate bleeding boundaries and support quantitative assessment of bleeding extent.

### 4.4. Comparative Analysis with State-of-the-Art Methods

Table 4 compares the proposed framework with representative and recent studies on WCE bleeding detection and segmentation. The proposed 2D-CNN achieved an accuracy of 97.47%, exceeding the 95.65% reported by Rani et al. (2022) [37]. Similarly, the proposed 3D-CNN achieved an accuracy of 97.98%, compared with 96.20% reported by Bordbar et al. (2023) [23], despite utilizing a substantially smaller dataset. For bleeding segmentation, the proposed U-Net achieved an accuracy of 97.25%, compared with 95.88% reported by Coelho et al. (2018) [19]. Although direct comparison should be interpreted with caution due to differences in datasets and evaluation protocols, the results demonstrate that the proposed models achieve competitive performance relative to recent state-of-the-art approaches.

### 4.5. Limitations of the Study

Despite the promising results, this study has several limitations. First, the datasets used in this work did not provide patient-level identifiers, preventing patient-wise data partitioning and introducing a potential risk of train–test correlation if multiple frames originated from the same examination. Second, the proposed models were evaluated using publicly available datasets with varying image characteristics and annotation protocols, which may affect generalizability to other clinical settings. Third, although the 3D-CNN achieved excellent performance, it was trained on a relatively small number of volumetric samples, and further validation on larger datasets is warranted. Finally, the bleeding-area estimation was derived from 2D segmentation masks and should be interpreted as a quantitative image-based measure rather than a direct estimate of the true three-dimensional volume or clinical severity of bleeding. Future work will focus on external multi-center validation, patient-wise evaluation, and integration of temporal bleeding progression analysis to further enhance clinical applicability.

## 5. Conclusions

This study presents a unified deep-learning framework for automated bleeding analysis in wireless capsule endoscopy. The proposed 2D-CNN provides fast and accurate frame-level bleeding detection, while the 3D-CNN exploits spatiotemporal information to improve sequence-level assessment. Complementing these classifiers, the U-Net enables precise pixel-level segmentation and quantitative bleeding-area estimation. Together, these models form an integrated pipeline capable of efficient frame filtering, reliable bleeding detection, and detailed lesion localization. The results demonstrate the feasibility of combining classification, segmentation, and quantitative analysis within a single framework, highlighting its potential to support clinical decision-making and improve the efficiency of wireless capsule endoscopy interpretation.

## Figures and Tables

**Table 1:** Summary of the Four WCE Datasets Used for Bleeding Detection and Segmentation.

	Dataset 1	Dataset 2	Dataset 3	Dataset 4
Bleeding	446	1131	113	1690
Non-bleeding	421	2164	113	2698
Total (frames)	867	3295	226	4388

**Table 2:** Performance of the 2D-CNN Model on Training data: Fold-Wise Metrics with Mean and SD.

Metric	Fold 1	Fold 2	Fold 3	Fold 4	Fold 5	Mean	SD
Loss	0.1571	0.1652	0.1710	0.1909	0.1528	0.1674	0.0150
Accuracy	0.9844	0.9716	0.9730	0.9645	0.9801	0.9747	0.0075
AUC	0.9982	0.9981	0.9977	0.9962	0.9990	0.9978	0.0010
Precision	0.9888	0.9737	0.9441	0.9697	0.9886	0.9730	0.0178
Recall	0.9706	0.9522	0.9890	0.9377	0.9596	0.9618	0.0197
F1-score	0.9796	0.9628	0.9660	0.9534	0.9739	0.9671	0.0093

**Table 3:** Performance of the 3D-CNN Model on Training Data: Fold-Wise Metrics with Mean and SD.

Metric	Fold 1	Fold 2	Fold 3	Fold 4	Fold 5	Mean	SD
Loss	0.0924	0.0413	0.0614	0.0674	0.0629	0.0651	0.0163
Accuracy	0.9784	0.9784	0.9784	0.9855	0.9783	0.9798	0.0029
AUC	0.9963	0.9994	0.9950	0.9977	0.9973	0.9971	0.0014
Precision	0.9726	0.9857	0.9857	0.9859	0.9722	0.9804	0.0066
Recall	0.9861	0.9718	0.9718	0.9859	0.9859	0.9803	0.0069
F1-score	0.9793	0.9787	0.9787	0.9859	0.9790	0.9803	0.0030

**Table 4:** Comparison of state-of-the-art WCE bleeding detection and segmentation methods with the proposed models.

Reference	Year	Task Type	WCE Frames	% Accuracy
Rani et al. [37]	2022	2D CNN + SVM	3895	95.65
Proposed (2D-CNN)	2025	Custom 2D CNN	4400	97.47
Bordbar et al. [21]	2023	3D-CNN classifier	14,691	96.20
Proposed (3D-CNN)	2025	Custom 3D CNN	867 volumes	97.98
Coelho et al. [17]	2018	U-Net segmentation	3895	95.88
Proposed (U-Net)	2025	U-Net segmentation	3295	97.25

## Data Availability

The datasets used in this study are publicly available and can be accessed through the references cited in the manuscript. Additional processed data are available from the corresponding author upon reasonable request.
